# Uncommon but Significant: A Case of Primary Lumbar Hernia Treated With Open Mesh Repair

**DOI:** 10.7759/cureus.96846

**Published:** 2025-11-14

**Authors:** Gautham Vignesh, Surendran Paramsivam, Manish Marlecha, Muthurenganathan P. L.

**Affiliations:** 1 General Surgery, Sri Ramachandra Institute of Higher Education and Research, Chennai, IND

**Keywords:** anatomy, hernia, hernioplasty, lumbar swelling, surgery

## Abstract

We present a case of a male in his late 50s who reported a painless swelling in the left lumbar region, noticed 15 days earlier. Examination revealed a soft, reducible swelling. Contrast-enhanced CT identified a 2.8 × 2.8 cm defect in the inferior lumbar triangle containing omentum and large bowel. An open mesh repair was performed, confirming the diagnosis intraoperatively. The patient had an uneventful recovery. The patient was discharged on postoperative day 3 after drain removal, and was reviewed one week after discharge on an outpatient basis, where it was found to be healthy and sutures removed. The patient was followed up one and two months after discharge, and no recurrence was noted. This case highlights the diagnostic challenge of lumbar hernias due to their subtle presentation and rarity.

## Introduction

Lumbar hernias are a rare and under-recognized abdominal wall defect. They develop from anatomical weak points in the posterior abdominal wall, most often in the superior (Grynfeltt-Lesshaft) or inferior (Petit) lumbar triangles [1]. Primary lumbar hernias, defined as those developed spontaneously but lack any history of previous trauma or surgical intervention, represent a very small proportion of the reported cases and pose a diagnostic and therapeutic concern for surgeons [2]. Lumbar hernias are uncommon, and therefore, the exact prevalence is hard to quantify. This lack of consensus leads to a limited standardization of management, given that most publications are single-case reports and small case series. Open repair with the use of tissue flaps is the cornerstone of treatment in human history. But recently, with breakthroughs in imaging and minimally invasive technologies, laparoscopic and robotic methods offer a new avenue for accurate defect detection, tension-free mesh insertion, and rapid postoperative healing [3]. Notwithstanding these developments, a variety of debates remain on the right surgical route, type of mesh, and method of fixation. Moreno-Egea et al. stressed that the decision regarding open and laparoscopic repair could be very specialized based on the size, location, and contents of the hernia, as well as a surgeon's experience [1]. Anatomical differences in the lumbar region further complicate diagnosis and repair planning. To provide a uniform approach to this rare condition, Heemskerk and coworkers presented a standardized treatment algorithm for primary lumbar hernias, underlining the need for detailed preoperative imaging and an individualized approach toward defect closure and reinforcement [4]. However, considering the low prevalence and variable reporting, a consensus on the topic is unclear. This present report contributes to the developing literature directed towards improving clinical understanding of the diagnosis and treatment of primary lumbar hernias. It aims to support the development of international diagnostic and treatment guidelines for the rare condition using in-depth written literature and discussion that may result.

## Case presentation

**Figure 1:**
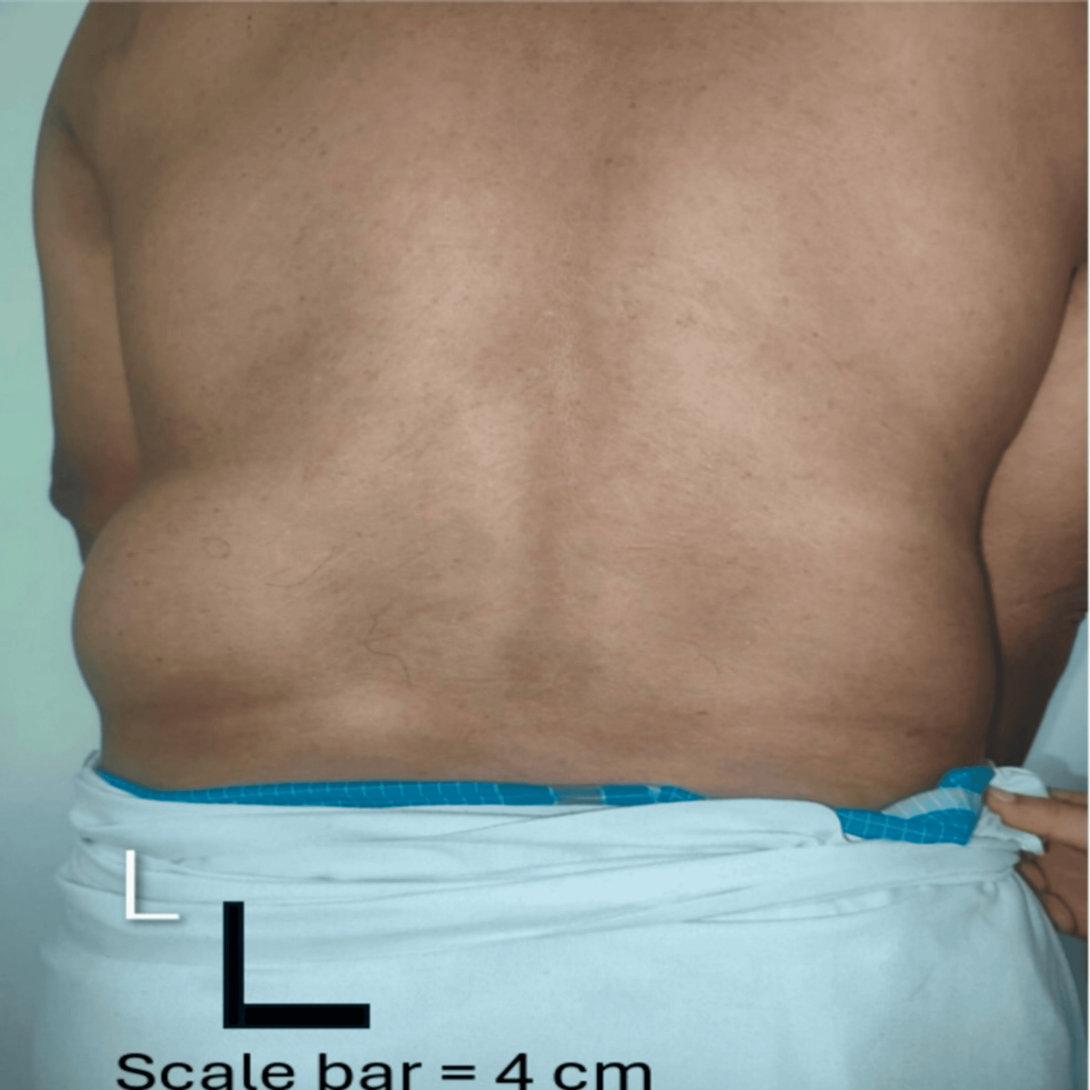
A 5 × 6 cm swelling located 4 cm below the costal margin and 1 cm above the iliac crest. L: left side

A 54-year-old male presented with a 15-day history of a gradually enlarging, painless swelling in the left flank. The swelling was noted to reduce in size upon lying in the prone position and increase during straining. There was no history of trauma, prior surgeries, or similar swellings. On physical examination, a soft, reducible swelling measuring approximately 5 × 6 cm was palpated in the left lumbar region, situated 4 cm below the costal margin and 1 cm above the iliac crest, extending anteriorly to the anterior axillary line (Video 1 and Figure 1).

**Video 1:** Lumbar hernia demonstrating a visible cough impulse.

**Figure 2:**
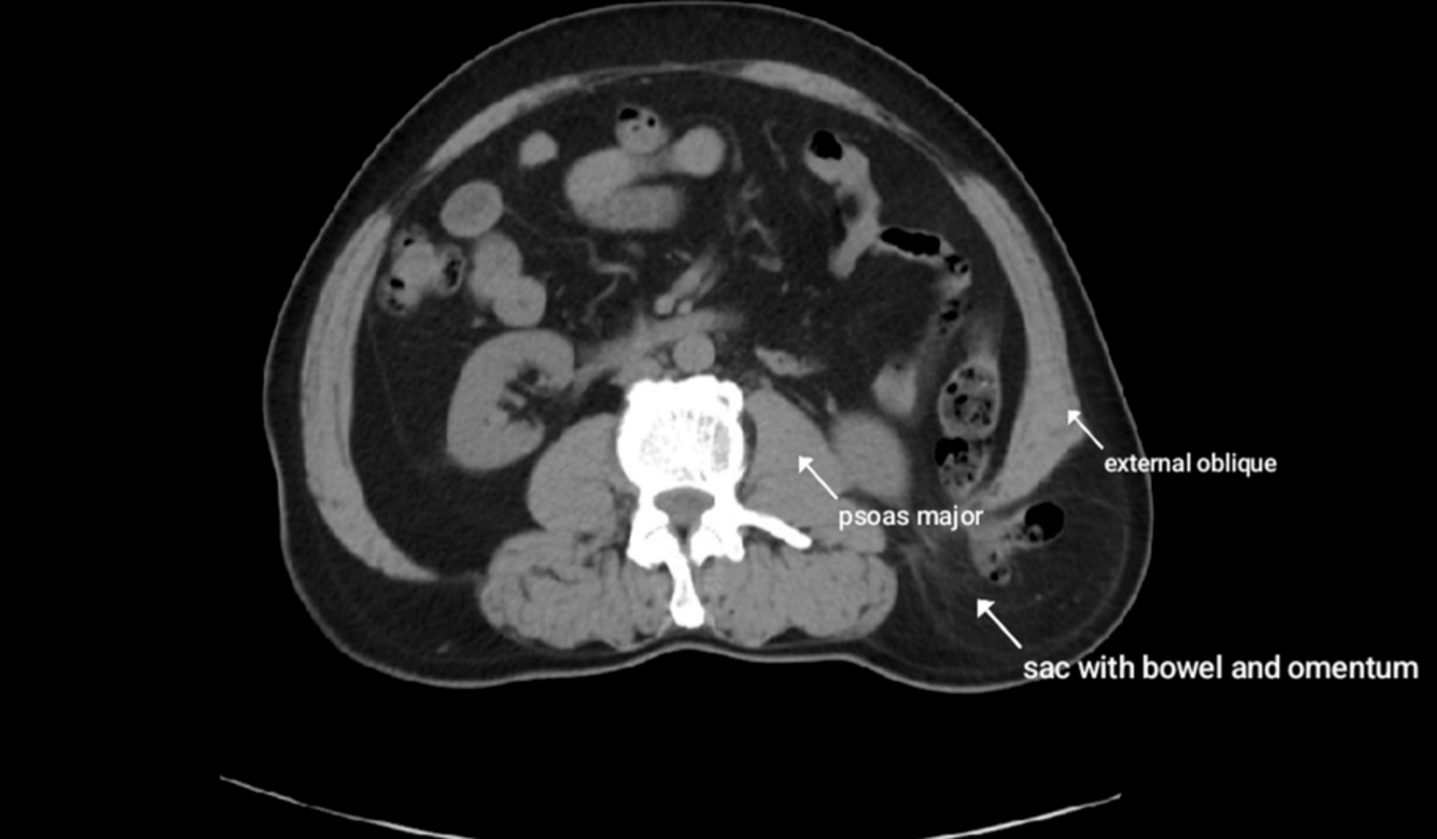
CT scan demonstrating an inferior lumbar hernia with omentum and bowel as content.

Contrast-enhanced computed tomography (CT) revealed a 2.8 × 2.8 cm defect in the inferior lumbar triangle, with herniation of preperitoneal fat, omentum, and a short segment of descending colon. The hernial sac measured 7.5 × 7.5 cm with an estimated volume of 151 cc, and the hernia-to-rectus defect ratio was calculated to be 4.2 (Figure 2).

**Figure 3:**
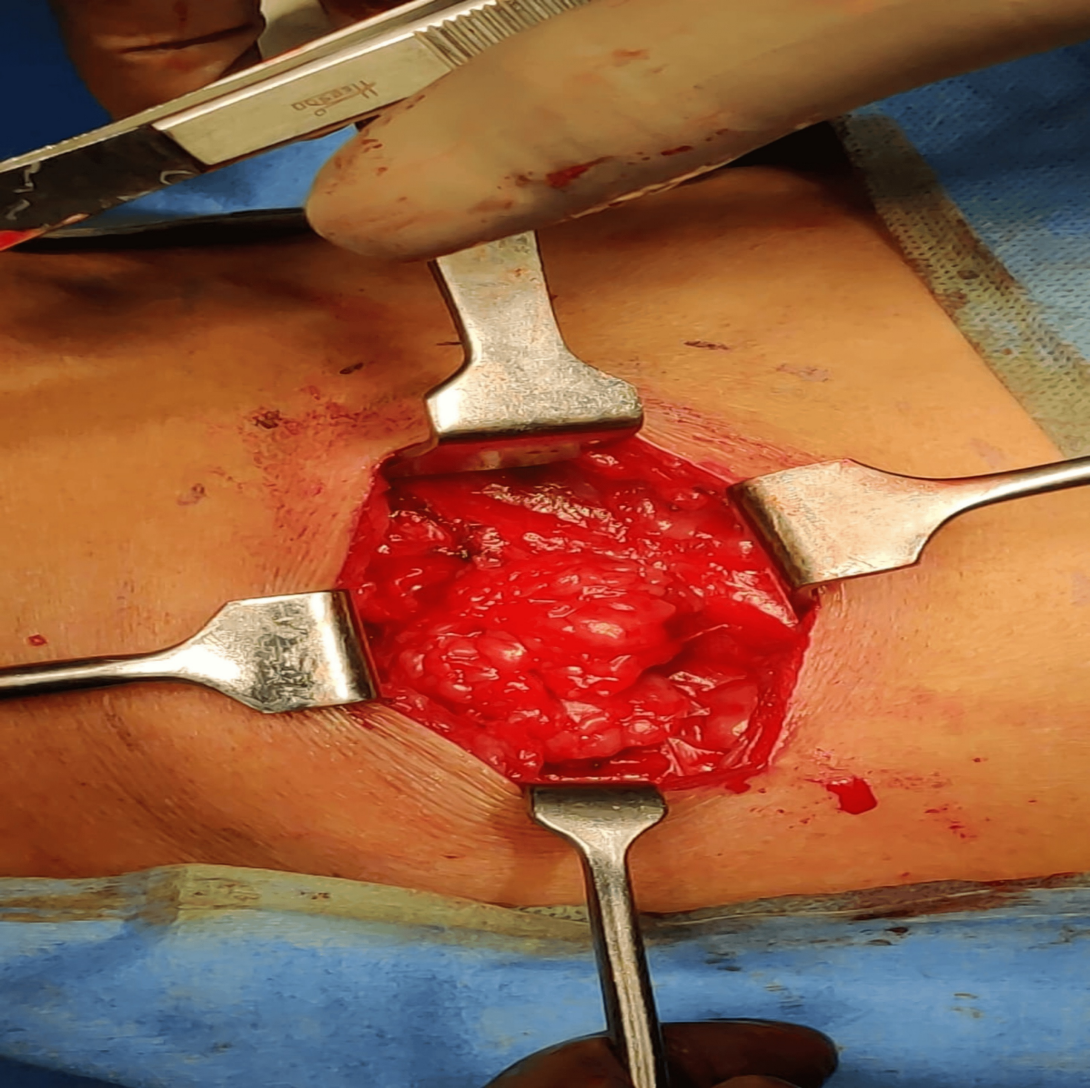
Lumbar hernia with omentum as content.

**Figure 4:**
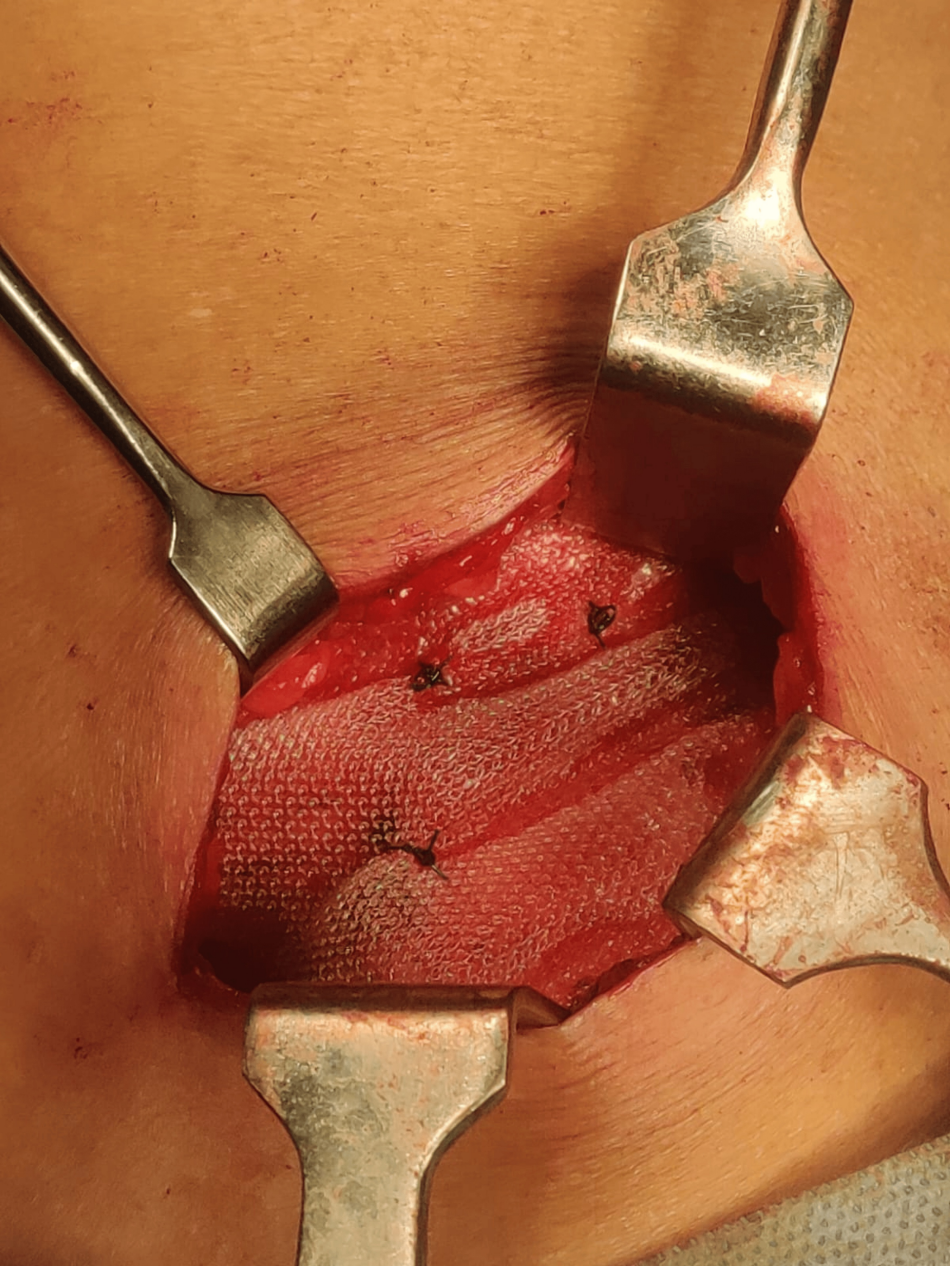
Hernial defect reinforced with a 15 × 15 cm Prolene mesh (Ethicon, Somerville, NJ, USA) after myofascial approximation.

The patient was taken up for elective open lumbar hernioplasty under general anesthesia. A lumbar incision was made on the left side, and dissection was performed layer by layer. The hernial sac was identified emerging from the inferior lumbar triangle, and the anatomical borders - latissimus dorsi posteriorly, external oblique anteriorly, and iliac crest inferiorly - were clearly delineated. Sharp and blunt dissection was used to isolate the sac, which contained preperitoneal fat and omentum. The contents were carefully reduced into the peritoneal cavity. The defect was closed using myofascial approximation. A 15 × 15 cm polypropylene mesh was trimmed, positioned over the defect, and anchored securely using interrupted 2-0 Vicryl sutures (Ethicon, Somerville, NJ, USA) to surrounding muscular and fascial structures. Hemostasis was meticulously achieved. A 16 Fr closed suction Romovac drain (Romsons, Agra, India) was placed in the subfascial plane, and the wound was closed in layers. The postoperative period was uneventful. The drain was removed on postoperative day 4, and the patient was discharged on day 5. At follow-up, the surgical site was healthy, with no signs of recurrence, and the patient had returned to normal daily activity (Video 2 and Figures 3-4).

**Video 2:** Description of the steps of surgery with explanation of anatomy and omentum seen as content.

## Discussion

Lumbar hernias are uncommon defects of the posterior abdominal wall, accounting for less than 2% of all abdominal wall hernias [1,2]. They are classified broadly into congenital (≈ 10-20%), primary (spontaneous), and secondary (post‑surgical or traumatic) types [3]. Congenital lumbar hernias often present in infancy and are frequently associated with musculoskeletal anomalies, especially as components of lumbo‑costovertebral syndromes. These may include vertebral malformations (e.g., hemivertebrae, scoliosis), rib defects (hypoplasia or fusion), pelvic asymmetries (e.g., iliac hypoplasia), and deficient posterior abdominal wall musculature (internal oblique, transversus abdominis, quadratus lumborum) [3]. In many such patients, additional orthopedic abnormalities (e.g., congenital hip dislocation, limb length discrepancy, clubfoot) may coexist, likely reflecting early mesodermal dysgenesis.

Primary lumbar hernias typically manifest in adulthood and are associated with contributory factors such as chronic straining, obesity, or degenerative muscle weakening, while secondary hernias occur after trauma or flank operations (e.g., nephrectomy, iliac crest graft harvesting) [4].

Anatomic defects most often occur through two zones of structural weakness: the superior (Grynfeltt-Lesshaft) triangle and the inferior (Petit’s) triangle. The superior triangle is delimited by the 12th rib (superior), quadratus lumborum (medial), and internal oblique (lateral), with transversalis fascia forming the floor; it is the most frequent hernia site. The inferior triangle is bordered by the iliac crest (inferior), external oblique (anterior), and latissimus dorsi (posterior), with internal oblique forming its floor. Herniation through the inferior triangle is relatively rare [5].

Clinically, lumbar hernias often present insidiously. A soft lumbar swelling exhibiting cough impulse and reducibility may be evident (especially in prone posture). Symptoms such as localized discomfort, back pain, or tenderness are common. In obese individuals, adiposity may mask the defect, leading to diagnostic confusion with lipomas, abscesses, or other soft‑tissue masses [6,7]. Imaging, particularly CT, is considered the gold standard, allowing precise delineation of the defect, hernia contents, and spatial relationships [8,9].

Timely diagnosis is imperative: reported rates of incarceration are approximately 25-30%, and strangulation in ~8-10%, potentially resulting in bowel obstruction, ischemia, or perforation [10]. Thus, early surgical planning is warranted. Reducible hernias may undergo elective repair, whereas incarcerated or strangulated cases demand emergent intervention. Historically, repairs via primary tissue closure or flap techniques had high recurrence and complication rates (hematoma, seroma). The advent of synthetic mesh revolutionized management, favoring tension‑free techniques (onlay, inlay, sublay). Among these, sublay (retro‑muscular or extraperitoneal) placement often yields better mesh integration and lower recurrence rates; however, onlay repair with myofascial approximation remains a viable option in small localized defects or in settings with limited resources.

In our case, a 54-year-old man with a 2.8 × 2.8 cm inferior lumbar (Petit’s) hernia containing omentum and bowel, we selected an open onlay mesh repair with primary myofascial closure. After reduction of the herniated contents and approximation of the muscular edges, a 15 × 15 cm polypropylene mesh was placed over the repair. This approach afforded direct exposure, tension mitigation through tissue re-approximation, and reinforced coverage. Advantages include surgical simplicity, shorter operative time, and reliable anatomical restoration in appropriately selected patients.

Laparoscopic approaches (e.g., transabdominal, totally extraperitoneal, or intraperitoneal onlay) offer the benefits of improved cosmesis, less postoperative pain, and faster recovery but demand higher technical skill and may be limited by distorted anatomy or suboptimal access for mesh fixation. Open onlay repair, while dependable, carries potential risks such as seroma, infection, hematoma, mesh complications, and chronic neuralgia; however, meticulous dissection, nerve preservation, dead-space obliteration, and judicious drain placement can help mitigate these issues. In our patient, the suction drain was removed on postoperative day 3, and the patient was discharged. The patient was followed up in the outpatient department, where the wound was healthy and the sutures were removed. He was able to resume day-to-day activities after one week. Recovery was uneventful, and no recurrence was noted during follow-up at one and two months.

Limitations

This report describes a single case with a relatively short follow-up period and without postoperative imaging confirmation of repair integrity. Further long-term studies or multi-institutional case series are warranted to validate the durability and outcomes of open onlay repair for primary lumbar hernias.

## Conclusions

This case highlights the clinical importance of understanding lumbar anatomy and customizing the surgical approach based on individual patient characteristics. The successful postoperative outcome reinforces that open onlay mesh repair with myofascial approximation remains a safe, effective, and reproducible technique, particularly in settings with limited resources or when anatomical landmarks are well-defined. By documenting this rare presentation and its favorable management outcome, the report adds valuable insight to the limited body of literature on primary lumbar hernias and serves as an educational reference for surgeons encountering similar cases.
